# Characterization of *Larix decidua* Mill. (Pinaceae) oleoresin’s essential oils composition using GC-MS

**DOI:** 10.3389/fpls.2023.1331894

**Published:** 2024-01-08

**Authors:** João V. C. Batista, Michelle Nonato de Oliveira Melo, Carla Holandino, Jakob Maier, Jörg Huwyler, Stephan Baumgartner, Fabio Boylan

**Affiliations:** ^1^ Hiscia Institute, Society for Cancer Research, Arlesheim, Switzerland; ^2^ Department of Pharmaceutical Sciences, Division of Pharmaceutical Technology, University of Basel, Basel, Switzerland; ^3^ Departamento de Fármacos e Medicamentos, Faculdade de Farmácia, Universidade Federal do Rio de Janeiro, Rio de Janeiro, Brazil; ^4^ Institute of Integrative Medicine, University of Witter/Herdecke, Witten, Germany; ^5^ Institute of Complementary and Integrative Medicine, University of Bern, Bern, Switzerland; ^6^ School of Pharmacy and Pharmaceutical Sciences, Trinity Biomedical Sciences Institute, Trinity Natural Products Research Centre, Trinity College Dublin, Dublin, Ireland

**Keywords:** larch, hydrodistillation, essential oil, oleoresin, gas chromatography, *Larix decidua*

## Abstract

**Introduction:**

Larch oleoresin has been described regarding several biological activities and medicinal applications, such as wound healing and treatment of ulcers, but little is known about its chemical composition.

**Material and methods:**

Eight oleoresins from *Larix decidua* Mill. obtained from four companies and one adulterated control were therefore investigated to determine their content of essential oils and to verify possible differences in their composition in relation to the harvest and manufacturing processes. Essential oils (EOs) were isolated by distillation and the yield was analysed.

**Results and discussion:**

The yield of EO varied among all samples. The yield of the pure larch samples covered a range of 7.8% to 15.5%. A higher yield (19.0%) was observed for adulterated control, which contained oleoresins from different Pinaceae trees. Age of samples had no impact on yield. However, there was a significant statistical variation (p<0.05) in the yields of the mid-summer oleoresins (>10%) compared to early or late summer (<10%), emphasising the importance of the time of collection. Samples were subsequently analysed by GC-MS. EO samples confirmed the presence of various chemical classes, such as monoterpenes, sesquiterpenes, and diterpenes. α-pinene was the compound with the highest concentrations (>50%), followed by β-pinene (>6%), D-limonene (>2.5%), α-terpineol (>0.9%), β-myrcene (>0.2%), and 3-carene (>0.05%). Samples were grouped using multivariate data analysis (MVDA) with respect to the chemical variation between the oleoresins’ EOs. The resulting four clusters were named low (low yield obtained for the samples), mixed (mixed oleoresin from different Pinaceae species, adulteration control), old (old oleoresin kept in the institute), and normal (other oleoresins) samples, each presenting distinct chemical biomarkers. There were considerable differences between site and time of collection. Essential oil yield did not always meet requirements as defined by the German Homeopathic Pharmacopoeia. In addition, adulterated or aged samples could be identified as compared to pure and fresh larch oleoresins.

**Conclusion:**

We conclude that larch oleoresin used for pharmaceutical applications has to be carefully analysed and standardised to guarantee reproducible product quality.

## 1 Introduction

Natural products have long been an essential source of new drugs against various diseases, leading to the discovery of several natural antibiotics, and are equally employed successfully as cancer therapeutics (Atanasov et al., 2021; Dzobo, 2022). Due to the complex mixture of different compounds, there is a potential for synergistic therapeutic effects (Atanasov et al., 2021). Essential oils (EOs) are aromatic and volatile substances extracted from various parts of plants, such as leaves, flowers, fruits, and even bark (Harrewijn et al., 2000; Tongnuanchan and Benjakul, 2014) and have been used for centuries for their therapeutic, cosmetic, and culinary properties (Figueiredo et al., 2008; Sharmeen et al., 2021). Among the diverse sources of EOs, conifers stand out as a remarkable botanical group (Franz and Novak, 2015).

*Larix decidua* Mill. (Pinaceae), commonly known as the European larch (Tropicos, 2023), is a deciduous (The_World_Flora, 2023), coniferous tree with delicate foliage, which occurs in the central (Alps) and eastern mountains of Europe (Da Ronch et al., 2016). Beyond its striking appearance and ecological significance, this tree holds a valuable secret within its resinous sap, known as oleoresin, commonly referred to as turpentine (Dietemann et al., 2019). This oleoresin, rich in useful compounds, has been employed in various industries, from traditional medicine to perfumery and beyond (Lagoni, 2012). Our recent review showed that a lot is known about the species’ phytochemical composition, but only little is known about the oleoresin’s composition and biological properties (Batista et al., 2022).

Plant extracts are complex mixtures of compounds and need to be analysed and identified to monitor the quality of the sample and its identity. In this work, we investigated the yield and differences in composition of the EOs in eight different larch oleoresin batches and in one adulterated sample used as control. Gas chromatography tandem mass spectrometry enabled the separation and identification of volatile organic compounds, which were grouped using multivariate data analysis to identify preparations, which comply with Pharmacopeia and product quality requirements.

## 2 Material and methods

### 2.1 Plant materials and reagents

Oleoresins of *Larix decidua* Mill. were obtained from 4 commercial companies: Brüder Unterweger (BU, Austria); Röper (R, Germany); Hänseler (H, Switzerland); and Schusser (S, Austria). **Table 1** lists sample codes, batches, place, and time of collection of each oleoresin used in this study.

**Table 1:** List of oleoresins analysed and their collection information.

Sample Code	Batch	Site of Collection	Collection Time
BU1	020156 Pos 1	South Tyrol, IT	May 2020
BU2	020156 Pos 2	South Tyrol, IT	August 2020
BU3	020156 Pos 3	East Tyrol, AT	September 2020
BU4	020156 Pos 4	South Tyrol, IT	June 2021
R3	2568501	Trentino, South Tyrol, IT	Summer 2019
H	2020.07.0801	nd	2018
Ha	403058	nd	nd
S	204601	Carinthia, AT	August 2020
BUVT	022968 Pos 1	nd	August 2022

Ha is an old oleoresin sample kept in our institute, dated 1994. BUVT, called Venice Turpentine, is a mixture of oleoresins from several species, such as *Larix decidua, Abies alba, Pinus pinaster* and *Picea excelsa*. nd = not declared / unknown.

### 2.2 Hydrodistillation

The essential oils (EOs) were obtained following the German Homeopathic Pharmacopeia method (HAB, 2014). The oleoresins were submitted to hydrodistillation in a 500 mL round flask with 200 mL of distilled water and boiling pebbles (sort A, ROTH) for 2 h, and a Clevenger-type apparatus was used (as in Ph. Eur. 2.8.12), with distillation at a rate of 3 mL/min. The procedure was performed using xylol (0.5 mL) in the graduation of the apparatus. The EO yield was determined by sight using the graduation of the apparatus as a subtraction of the final volume and the initial one (0.5 mL). The distillates were stored in a glass-sealed vial at 4°C until chromatographic analysis.

### 2.3 GC-MS analyses

The GC-MS analysis was performed in a Shimadzu GCMS-QP2010 SE (Shimadzu, Ireland). A capillary column ZB-5Plus of 30 m x 0.25 mm x 0.25 µm was used for the separation. The GC parameters were as follows: the carrier gas was highly pure helium with a 1 mL/min flow rate. The inlet temperature was 250°C with a split ratio of 20:1 and the pressure was 49.7 kPa. The column oven temperature was initially set at 40°C for 1 min, and then ramped to 290°C at 5°C/min, and kept at 290°C for 5 min.

MS parameters were as follows: data were acquired in the electron impact (EI) mode, using the full scan mode from *m*/*z* 40 to 750. The ion source and interface temperatures were 200°C and 300°C, respectively. The identification of the volatile compounds was based on a comparison of their GC retention time and mass spectra with the retention index of *n*-saturated alkanes and the reference spectra from the US National Institute of Standards and Technology (NIST, 2023). Data was analysed by Shimadzu LabSolution Postrun software.

### 2.4 Statistical analysis

The GC-MS data of the volatiles was analysed using multivariate data analysis (MVDA) to group samples with respect to the chemical variation between the nine oleoresins’ essential oils. Relative abundance (% area) of each compound were calculated based on the ratio between the peak area of each compound and the sum of all integrated compounds. The data of the MVDA was exported to Metaboanalyst 5.0 web server to observe how the samples are clustered. Firstly, unsupervised analysis was done by hierarchical cluster analysis (HCA) evaluated by Euclidian distance dissimilarity using the aggregation criterion of Ward’s method and by principal component analysis (PCA). Afterwards, a supervised analysis was done by the partial least squares discriminant analysis (PLS-DA) to examine the separation between the groups and to better comprehend the variables responsible for classification (Melo et al., 2022). Analysis of variance (ANOVA) followed by Tukey’s *post hoc* test was performed, using the same web server for the box-plot analysis. Differences were considered significant when *p*<0.05.

## 3 Results and discussion

### 3.1 Distillation process

The distillation process using a Clevenger-type apparatus allowed not only to obtain the EOs but also provided the yield of EO, an important information for the oleoresin’s quality control. **Table 2** shows the essential oils yield obtained for each of the nine samples of oleoresin.

**Table 2:** Yield of essential oil in each sample of oleoresin after distillation (mean ± SD, n=3).

	BUVT	Ha	H	S	BU1	BU2	BU3	BU4	R3
**Amount of resin (g)**	2.0	2.0	2.0	2.0	2.0	2.0	2.0	2.0	2.0
**Volume of EO (mL)**	0.38 ± 0.02	0.27 ± 0.00	0.29 ± 0.01	0.31 ± 0.01	0.16 ± 0.02	0.24 ± 0.01	0.18 ± 0.02	0.21 ± 0.01	0.25 ± 0.01
**Yield (v/w)**	19.0 ± 0.8%	13.5 ± 0.0%	14.3 ± 0.6%	15.5 ± 0.4%	7.8 ± 1.0%	12.0 ± 0.4%	8.8 ± 0.9%	10.7 ± 0.6%	12.3 ± 0.2%

**Figure 1:**
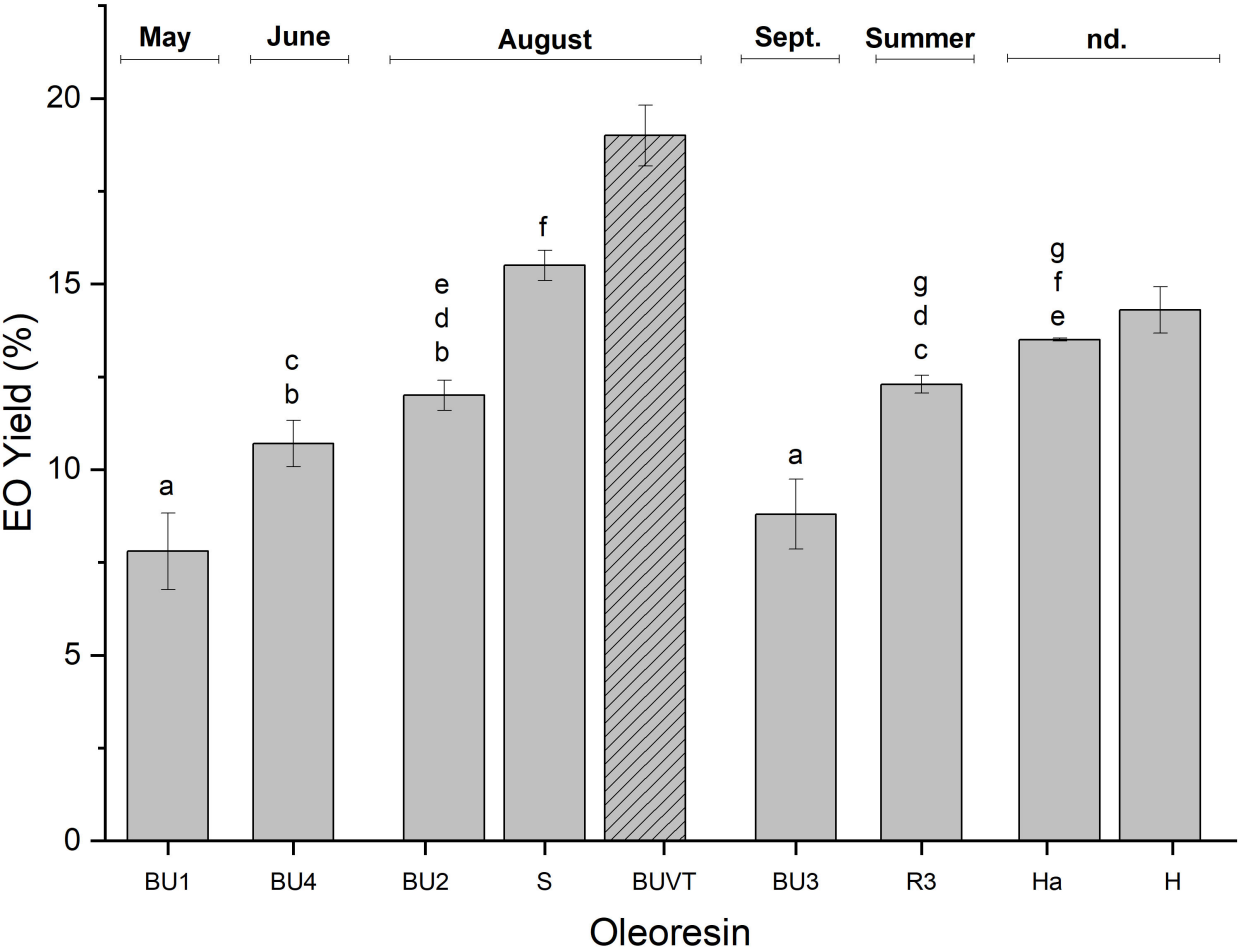
The essential oil yield (% v/w) of *Larix decidua* oleoresins at different collection times (values are means **±** SD, n=3). BUVT: adulteration control. According to Pharmacopeia requirements, a minimum yield of 10% has to be reached. (A-G): there were no statistically significant differences between samples with the same letter (p>0.05). Samples with no letters (BUVT, H) were statistically different to all other samples. nd, not declared.

Analysis of the yield of different oleoresins EO showed statistical differences (p<0.05) between harvesting times, and within the same collected month. The highest content of essential oil was found in the mixed oleoresin (BUVT, adulteration control, 19%), followed by S (15.5%) and H (14.3%; **Figure 1**), the two first collected in August and the last was not declared. The lowest amount of EO was obtained from BU1 (7.8%) and BU3 (8.8%), collected in May and September, respectively. BU1 and BU3 presented less than 10% yield, which is below the specification of the Larch oleoresin monography with its range between 10 to 20% (v/w) (HAB, 2014). Therefore, all the oleoresins were within the specified range, except BU1 and BU3. Similar EO yield was found in the literature for oleoresins from *Larix* sp. (15-20%) (Weissmann and Reck, 1987), *Pinus merkusii* (19.6-13.6%) (Sukarno et al., 2015), *Pinus roxburghii* (16% w/w) (Ayub et al., 2022), *Pinus patula* (14.55%) and *Pinus oocarpa* (14.40%) (Sarria-Villa et al., 2021), and *Pistacia atlantica* L. (10% w/w) (Mohtashami et al., 2023). Oleoresins BU1 and BU3 were obtained in early (May) and late (September) summer (**Table 1**), respectively, and according to Karimian et al. (2020), EO yield (w/w) from *Ferula asa-foetida* oleo-gum-resin was higher in July (9.1 ± 1.53%) and lower in October (7.4 ± 0.85%). It supports our findings that early (May) and late (September) summer provided less EO than high (August) summer. In addition, oleoresin production is higher in midsummer (Karimian et al., 2020) and might influence EO yield.

Variability of essential oil yield in oleoresin is a complex phenomenon influenced by a combination of biotic and abiotic factors, such as plant source, environmental conditions, harvesting and extraction methods, seasonal variations, storage, and genetics (Rasgado-Bonilla et al., 2016; Yu et al., 2020). European larch grows on different sites, such as locations in Poland and the Alps. Its distribution ranges from 180 m to 2500 m in altitude (Da Ronch et al., 2016). The investigated samples were collected from South Tyrol (Italy), East Tyrol (Austria), Carinthia (Austria), and unknown locations. The altitude in these areas varies between 600 and 3900 m, and the precise site of collection and the altitude should be known for a better understanding of the obtained results. The influence of altitude on the oleoresin and EO were investigated by Sukarno et al. (2015), who described a positive correlation between the essential oil yield and elevation. Environmental influences need further investigation for *Larix decidua* oleoresin and EO yield and their bioactive constituents.

### 3.2 Comparative study of the chemical compositions of the essential oils

GC-MS analyses of the EOs of *L. decidua* collected at different times and from different companies as well as those of the adulteration control (BUVT) resulted in the identification of 74 volatile organic compounds (VOC, including 60 identified and 14 unidentified compounds, **Table 3**). These VOCs were found in one or more oleoresins EOs, which represent 96.60% to 100% of the total oils. The peak area of each VOC was measured as a function of its relative abundance to their elution in the ZB-5Plus column. The retention indices (RI) and the average percentage of each VOC in each of the 9 oleoresins are summarised in **Table 3**. Representative chromatographic profiles obtained for *L. decidua* EOs are shown in **Supplementary Figure 1**. Monoterpenes (80.98 ± 3.78% – 94.67 ± 1.61%), sesquiterpenes (2.25 ± 0.87% – 13.80 ± 0.80%) and diterpenes (0.00% – 3.28 ± 0.61%) were identified in the EOs obtained from the Larch oleoresins. Of the total GC-MS eluted compounds, monoterpenes hydrocarbons (73.48 ± 2.98% – 88.79 ± 1.02%) and oxygenated monoterpenes (4.09 ± 0.08% – 7.50 ± 0.81%) were the most abundant, with α-pinene (2, number in **Table 3**) (54.12 ± 2.16% – 72.39 ± 0.70%) and β-pinene (**7**) (6.30 ± 0.18% – 15.53 ± 0.17%) always as the dominant volatiles. In addition, D-limonene (14) (2.87 ± 0.01% – 5.92 ± 0.26%), α-terpineol (27) (1.09 ± 0.13% – 2.35 ± 0.29%), camphene (3) (1.02 ± 0.08% – 1.36 ± 0.01%), β-myrcene (**8**) (0.22 ± 0.04% – 2.67 ± 0.17%), and 3-carene (10) (0.07 ± 0.00% – 3.16 ± 0.07%), were the predominant volatiles for all the oleoresins EOs.

**Table 3:** Chemical composition in percentage (%) of the investigated Larix decidua oleoresin essential oils (as well as those from the adulteration control BUVT) obtained by gas chromatography coupled with mass spectrometry (mean±SD; n=3).

Number	RT	Compound	Formula	Descriptor^a^	RI		BUVT	Ha	H	S	BU1	BU2	BU3	BU4	R3
					Lit.^b^	Exp.^c^	Mean ± SD	Mean ± SD	Mean ± SD	Mean ± SD	Mean ± SD	Mean ± SD	Mean ± SD	Mean ± SD	Mean ± SD
1	3.78	α thujene	C_10_H_16_	wood, green, herb	932.00	932.06	-	0.21 ± 0.03	0.14 ± 0.03	0.10 ± 0.02	0.39 ± 0.05	0.17 ± 0.04	0.40 ± 0.02	0.31 ± 0.01	0.11 ± 0.02
2	3.92	α-pinene	C_10_H_16_	pine, turpentine	940.00	941.49	63.57 ± 0.61	72.39 ± 0.70	64.42 ± 1.24	67.94 ± 0.22	54.12 ± 2.16	65.49 ± 0.54	57.12 ± 1.89	65.30 ± 3.54	66.69 ± 1.07
3	4.14	camphene	C_10_H_16_	camphor	956.00	956.79	1.36 ± 0.01	1.32 ± 0.02	1.19 ± 0.08	1.24 ± 0.05	1.02 ± 0.08	1.17 ± 0.05	1.09 ± 0.03	1.18 ± 0.04	1.27 ± 0.04
4	4.24	dehydrosabinene	C_10_H_14_	n.s.	960.00	962.97	0.13 ± 0.03	0.17 ± 0.01	-	-	-	-	-	-	-
5	4.49	compound 1	–	–	–	980.12	-	0.18 ± 0.01	-	-	-	-	-	-	-
6	4.50	sabinene	C_10_H_16_	pepper, turpentine, wood	978.00	980.55	-	0.05 ± 0.00	0.37 ± 0.01	0.62 ± 0.04	0.13 ± 0.02	0.31 ± 0.01	0.15 ± 0.02	0.22 ± 0.03	0.32 ± 0.02
7	4.58	β-pinene	C_10_H_16_	pine, resin, turpentine	983.00	986.34	15.53 ± 0.17	6.34 ± 0.09	7.28 ± 0.35	7.11 ± 0.16	6.30 ± 0.18	7.91 ± 0.11	6.65 ± 0.23	7.48 ± 0.06	7.71 ± 0.21
8	4.72	β-myrcene	C_10_H_16_	balsamic, must, spice	992.00	995.74	0.93 ± 0.03	0.22 ± 0.04	2.59 ± 0.19	2.67 ± 0.17	1.99 ± 0.09	1.95 ± 0.02	2.22 ± 0.06	2.00 ± 0.08	2.17 ± 0.08
9	5.04	α-phellandrene	C_10_H_16_	turpentine, mint, spice	1013.00	1012.36	0.11 ± 0.03	0.08 ± 0.02	0.13 ± 0.00	0.14 ± 0.01	0.11 ± 0.04	0.08 ± 0.02	0.19 ± 0.04	0.08 ± 0.02	0.18 ± 0.09
10	5.15	3-carene	C_10_H_16_	lemon, resin	1013.00	1017.81	0.07 ± 0.00	3.16 ± 0.07	2.24 ± 0.22	2.51 ± 0.23	1.99 ± 0.10	1.10 ± 0.03	2.19 ± 0.03	1.50 ± 0.04	0.98 ± 0.05
11	5.24	1,4-cineole	C_10_H_16_O	spice	1016.00	1022.53	0.06 ± 0.01	-	-	-	-	-	-	-	-
12	5.26	α-terpinene	C_10_H_16_	lemon	1019.00	1023.38	0.08 ± 0.00	0.06 ± 0.01	-	0.13 ± 0.00	0.17 ± 0.00	-	0.22 ± 0.06	0.06 ± 0.00	0.21 ± 0.11
13	5.40	*o*-cymene	C_10_H_14_	n.s.	1029.00	1030.07	0.25 ± 0.02	0.71 ± 0.02	0.83 ± 0.03	0.91 ± 0.07	1.11 ± 0.12	0.71 ± 0.07	0.82 ± 0.06	0.80 ± 0.15	0.66 ± 0.20
14	5.48	D-limonene	C_10_H_16_	lemon, orange	1034.00	1034.46	5.13 ± 0.05	2.87 ± 0.01	5.29 ± 0.38	5.06 ± 0.31	5.56 ± 0.04	4.76 ± 0.03	5.91 ± 0.22	5.07 ± 0.09	5.92 ± 0.26
15	6.09	γ-terpinene	C_10_H_16_	gasoline, turpentine	1066.00	1064.40	0.08 ± 0.01	0.11 ± 0.05	0.12 ± 0.06	0.13 ± 0.02	0.16 ± 0.07	0.13 ± 0.03	0.37 ± 0.05	0.14 ± 0.01	0.35 ± 010
16	6.73	α-terpinolene	C_10_H_16_	n.s.	1089.00	1095.99	0.87 ± 0.09	0.33 ± 0.02	0.35 ± 0.15	0.42 ± 0.19	0.58 ± 0.27	0.37 ± 0.08	0.91 ± 0.10	0.44 ± 0.07	0.85 ± 0.27
17	6.99	α-pinene oxide	C_10_H_16_O	n.s.	1103.00	1107.28	0.07 ± 0.01	0.15 ± 0.00	0.36 ± 0.07	0.46 ± 0.21	0.29 ± 0.05	0.36 ± 0.07	0.16 ± 0.07	0.29 ± 0.12	0.28 ± 0.00
18	7.17	compound 2	–	–	–	1114.89	-	-	0.20 ± 0.05	0.24 ± 0.09	-	0.17 ± 0.00	-	0.14 ± 0.01	-
19	7.32	fenchol	C_10_H_18_O	camphor	1119.00	1121.18	0.25 ± 0.00	0.20 ± 0.03	0.25 ± 0.04	0.30 ± 0.05	0.43 ± 0.04	0.30 ± 0.02	0.40 ± 0.04	0.36 ± 0.06	0.42 ± 0.04
20	7.93	isopinocarveol	C_10_H_16_O	flower	1141.00	1147.11	0.20 ± 0.01	0.51 ± 0.02	0.19 ± 0.05	0.17 ± 0.09	0.14 ± 0.06	0.17 ± 0.02	-	0.15 ± 0.03	-
21	8.04	*cis*-verbenol	C_10_H_16_O	n.s.	1143.00	1151.63	0.09 ± 0.04	0.38 ± 0.01	0.35 ± 0.13	0.47 ± 0.11	0.35 ± 0.15	0.25 ± 0.03	0.22 ± 0.05	0.29 ± 0.08	0.23 ± 0.05
22	8.15	camphene hydrate	C_10_H_18_O	n.s.	1148.00	1156.27	0.15 ± 0.02	0.21 ± 0.11	-	-	0.17 ± 0.06	0.16 ± 0.01	0.12 ± 0.06	0.11 ± 0.03	0.14 ± 0.03
23	8.55	α-phellandren-8-ol	C_10_H_16_O	must, camphor	1170.00	1173.23	0.35 ± 0.03	0.58 ± 0.12	0.34 ± 0.06	0.31 ± 0.00	0.47 ± 0.06	0.32 ± 0.03	0.50 ± 0.06	0.38 ± 0.06	0.47 ± 0.01
24	8.82	terpinen-4-ol	C_10_H_18_O	turpentine, nutmeg, must	1184.00	1184.56	0.20 ± 0.00	0.72 ± 0.09	0.77 ± 0.07	0.83 ± 0.02	1.30 ± 0.06	0.77 ± 0.05	1.19 ± 0.10	0.89 ± 0.10	0.76 ± 0.04
25	8.98	*p*-cymen-8-ol	C_10_H_14_O	citrus, must	1189.00	1191.16	0.11 ± 0.03	0.18 ± 0.04	0.21 ± 0.03	0.15 ± 0.02	0.14 ± 0.04	0.09 ± 0.04	-	0.14 ± 0.04	0.08 ± 0.00
26	9.03	compound 3	–	–	–	1193.42	-	-	0.20 ± 0.04	0.09 ± 0.02	0.10 ± 0.03	0.12 ± 0.06	-	0.15 ± 0.01	-
27	9.13	α-terpineol	C_10_H_18_O	oil, anise, mint	1197.00	1197.44	2.17 ± 0.03	1.09 ± 0.13	1.42 ± 0.08	1.51 ± 0.03	2.35 ± 0.29	1.72 ± 0.13	2.14 ± 0.12	1.79 ± 0.14	1.86 ± 0.16
28	9.31	myrtenal	C_10_H_14_O	spice	1196.00	1204.66	0.20 ± 0.02	0.38 ± 0.07	0.45 ± 0.05	0.36 ± 0.13	0.31 ± 0.05	0.31 ± 0.05	0.32 ± 0.05	0.26 ± 0.11	0.15 ± 0.11
29	9.63	verbenone	C_10_H_14_O	n.s.	1214.00	1217.57	-	0.20 ± 0.03	0.18 ± 0.07	0.11 ± 0.00	-	0.22 ± 0.00	-	0.17 ± 0.00	-
30	9.86	*trans*-carveol	C_10_H_16_O	caraway, solvent	1223.00	1226.62	-	0.16 ± 0.05	-	-	-	-	-	-	-
31	10.19	thymol methyl ether	C_11_H_16_O	n.s.	1235.00	1239.50	-	0.53 ± 0.05	0.51 ± 0.03	0.51 ± 0.08	0.46 ± 0.03	0.31 ± 0.03	0.40 ± 0.03	0.27 ± 0.06	0.24 ± 0.02
32	11.53	bornyl acetate	C_12_H_20_O	must, camphor	1289.00	1292.76	0.26 ± 0.02	0.27 ± 0.06	0.30 ± 0.05	0.31 ± 0.05	0.37 ± 0.08	0.24 ± 0.01	0.31 ± 0.04	0.20 ± 0.05	0.12 ± 0.01
33	11.83	compound 4	–	–	–	1304.96	-	-	0.40 ± 0.10	0.48 ± 0.27	0.22 ± 0.01	0.38 ± 0.06	0.12 ± 0.02	0.26 ± 0.13	0.29 ± 0.00
34	12.06	compound 5	–	–	–	1313.83	-	-	0.19 ± 0.03	0.38 ± 0.00	-	0.13 ± 0.08	-	0.12 ± 0.04	-
35	12.21	compound 6	–	–	–	1320.08	-	-	0.26 ± 0.05	0.31 ± 0.21	0.14 ± 0.06	0.20 ± 0.09	-	0.17 ± 0.08	0.20 ± 0.00
36	12.45	compound 7	–	–	–	1329.30	-	-	0.12 ± 0.07	0.27 ± 0.00	-	0.19 ± 0.04	-	-	-
37	12.64	compound 8	–	–	–	1337.04	-	-	-	-	0.15 ± 0.05	-	-	-	-
38	12.83	δ-elemene	C_15_H_24_	n.s.	1339.00	1344.72	-	-	-	-	0.24 ± 0.02	-	0.20 ± 0.03	-	-
39	12.97	compound 9	–	–	–	1349.92	-	-	-	0.12 ± 0.02	0.15 ± 0.06	0.14 ± 0.06	-	0.23 ± 0.04	0.08 ± 0.00
40	13.11	α-terpinyl acetate	C_12_H_20_O_2_	wax	1351.00	1355.50	-	0.46 ± 0.04	0.67 ± 0.10	0.45 ± 0.08	0.71 ± 0.09	0.39 ± 0.00	0.60 ± 0.08	0.37 ± 0.08	0.35 ± 0.02
41	13.14	α-cubebene	C_15_H_24_	herb, wax	1354.00	1356.74	-	-	-	-	0.40 ± 0.04	0.11 ± 0.04	0.32 ± 0.03	0.17 ± 0.07	-
42	13.26	α-longipinene	C_15_H_24_	n.s.	1358.00	1361.78	0.38 ± 0.02	-	-	-	-	-	-	-	-
43	13.65	cyclosativene	C_15_H_24_	n.s.	1368.00	1377.26	-	0.10 ± 0.03	0.12 ± 0.06	-	0.24 ± 0.04	0.16 ± 0.05	0.23 ± 0.01	0.14 ± 0.03	0.16 ± 0.01
44	13.74	ylangene	C_15_H_24_	n.s.	1373.00	1380.88	0.17 ± 0.05	0.16 ± 0.05	0.17 ± 0.07	-	0.35 ± 0.04	0.14 ± 0.05	0.28 ± 0.01	0.15 ± 0.06	0.14 ± 0.04
45	13.85	copaene	C_15_H_24_	wood, spice	1380.00	1385.06	0.36 ± 0.05	-	0.15 ± 0.03	-	0.18 ± 0.03	-	0.24 ± 0.03	0.14 ± 0.06	0.08 ± 0.03
46	14.23	β-elemene	C_15_H_24_	herb, wax, fresh	1393.00	1400.23	0.10 ± 0.01	-	-	-	0.18 ± 0.06	-	0.16 ± 0.04	-	-
47	14.33	compound 10	–	–	–	1404.18	-	-	-	-	0.10 ± 0.04	-	-	-	-
48	14.64	longifolene	C_15_H_24_	n.s.	1418.00	1416.85	2.96 ± 0.40	0.15 ± 0.02	0.15 ± 0.05	-	0.24 ± 0.05	-	0.27 ± 0.02	0.15 ± 0.05	0.07 ± 0.00
49	14.95	β-caryophyllene	C_15_H_24_	wood, spice	1431.00	1429.86	2.64 ± 0.34	0.13 ± 0.02	0.23 ± 0.08	0.23 ± 0.05	0.99 ± 0.01	0.52 ± 0.04	0.84 ± 0.02	0.54 ± 0.09	0.58 ± 0.05
50	15.24	γ-elemene	C_15_H_24_	green, wood, oil	1439.00	1441.75	-	0.16 ± 0.03	0.35 ± 0.06	0.12 ± 0.04	2.98 ± 0.30	0.63 ± 0.02	2.53 ± 0.14	1.32 ± 0.18	0.60 ± 0.05
51	15.78	humulene	C_15_H_24_	wood	1462.00	1463.83	0.46 ± 0.06	-	0.11 ± 0.04	-	0.47 ± 0.04	0.26 ± 0.02	0.42 ± 0.03	0.25 ± 0.08	0.26 ± 0.02
52	16.31	γ-muurolene	C_15_H_24_	wood	1480.00	1485.34	-	0.44 ± 0.02	0.48 ± 0.13	0.19 ± 0.04	1.39 ± 0.15	0.57 ± 0.04	1.14 ± 0.06	0.73 ± 0.12	0.33 ± 0.03
53	16.44	germacrene D	C_15_H_24_	wood, spice	1485.00	1490.74	-	0.16 ± 0.08	1.04 ± 0.16	0.81 ± 0.38	0.84 ± 0.13	1.16 ± 0.20	1.03 ± 0.11	0.87 ± 0.19	1.72 ± 0.08
54	16.75	γ-amorphene	C_15_H_24_	n.s.	1495.00	1503.69	-	0.19 ± 0.00	0.22 ± 0.03	0.08 ± 0.01	0.49 ± 0.05	0.26 ± 0.04	0.45 ± 0.03	0.25 ± 0.07	0.15 ± 0.03
55	16.86	α-muurolene	C_15_H_24_	wood	1505.00	1508.46	-	-	0.18 ± 0.04	-	0.53 ± 0.05	0.19 ± 0.04	0.45 ± 0.02	0.24 ± 0.05	0.13 ± 0.02
56	17.05	compound 11	–	–	–	1516.28	-	0.12 ± 0.02	0.08 ± 0.02	-	0.15 ± 0.02	0.14 ± 0.04	0.28 ± 0.04	0.12 ± 0.07	0.08 ± 0.03
57	17.16	butylated hydroxytoluene	C_15_H_24_O	n.s.	1514.00	1521.02	0.23 ± 0.03	0.21 ± 0.05	0.21 ± 0.03	0.15 ± 0.07	0.27 ± 0.09	0.48 ± 0.01	0.94 ± 0.03	0.58 ± 0.18	0.07 ± 0.01
58	17.24	γ-cadinene	C_15_H_24_	wood	1515.00	1524.60	-	0.37 ± 0.00	0.44 ± 0.09	-	1.11 ± 0.18	-	-	-	-
59	17.27	compound 12	–	–	–	1525.78	-	-	-	0.26 ± 0.02	-	-	-	-	-
60	17.40	δ-cadinene	C_15_H_24_	thyme, medicine, wood	1524.00	1531.32	0.46 ± 0.07	0.47 ± 0.04	0.43 ± 0.07	0.17 ± 0.05	1.44 ± 0.07	0.58 ± 0.03	1.38 ± 0.07	0.76 ± 0.15	0.32 ± 0.09
61	17.69	α-cadinene	C_15_H_24_	wood; wood	1539.00	1543.75	0.14 ± 0.00	0.25 ± 0.07	0.13 ± 0.05	-	0.33 ± 0.09	0.16 ± 0.07	0.33 ± 0.09	0.19 ± 0.06	0.49 ± 0.05
62	17.88	selina-3,7(11)-diene	C_15_H_24_	n.s.	1542.00	1552.06	-	0.16 ± 0.06	-	-	0.28 ± 0.03	0.16 ± 0.09	0.25 ± 0.02	0.12 ± 0.05	0.10 ± 0.00
63	18.26	germacrene B	C_15_H_24_	wood, earth, spice	1569.00	1568.19	-	0.36 ± 0.05	0.82 ± 0.05	0.55 ± 0.17	0.35 ± 0.00	0.75 ± 0.07	0.39 ± 0.06	0.40 ± 0.05	0.75 ± 0.07
64	18.88	caryophyllene oxide	C_15_H_24_O	herb, sweet, spice	1583.00	1594.48	0.08 ± 0.02	-	-	-	0.10 ± 0.02	-	-	-	-
65	20.15	τ-cadinol	C_15_H_26_O	herb	1648.00	1650.97	-	-	0.19 ± 0.09	0.16 ± 0.02	0.23 ± 0.08	0.24 ± 0.06	0.18 ± 0.06	0.18 ± 0.07	0.20 ± 0.00
66	20.43	α-cadinol	C_15_H_26_O	herb, wood	1662.00	1663.83	-	-	-	-	0.16 ± 0.04	0.17 ± 0.01	0.22 ± 0.06	0.08 ± 0.03	0.13 ± 0.00
67	21.71	compound 13	–	–	–	1722.20	-	-	0.17 ± 0.03	0.07 ± 0.00	-	-	-	-	-
68	26.25	cembrene	C_20_H_32_	n.s.	1939.00	1943.62	-	-	-	-	0.18 ± 0.03	0.07 ± 0.00	0.16 ± 0.07	-	-
69	27.49	isopimaradiene	C_20_H_32_	n.s.	1969.00	2007.45	-	0.08 ± 0.04	0.16 ± 0.06	-	0.18 ± 0.03	0.14 ± 0.01	0.13 ± 0.05	-	0.15 ± 0.00
70	28.62	13-epimanool	C_20_H_34_O	n.s.	2057.00	2068.39	-	1.53 ± 0.22	1.68 ± 0.70	1.04 ± 0.23	2.32 ± 0.53	1.60 ± 0.34	1.50 ± 0.74	1.26 ± 0.39	0.95 ± 0.43
71	31.61	isopimarinal	C_20_H_30_O	n.s.	2222.00	2236.77	-	0.19 ± 0.02	-	0.10 ± 0.02	0.24 ± 0.02	0.15 ± 0.04	0.25 ± 0.08	0.11 ± 0.05	0.13 ± 0.00
72	31.85	palustrinal	C_20_H_30_O	n.s.	2245.00	2250.68	-	0.28 ± 0.07	-	-	0.18 ± 0.06	0.15 ± 0.00	0.29 ± 0.18	0.14 ± 0.08	0.16 ± 0.06
73	32.40	dehydroabietal	C_20_H_28_O	n.s.	2263.00	2282.89	-	0.14 ± 0.03	0.17 ± 0.02	0.10 ± 0.03	0.18 ± 0.05	0.21 ± 0.01	0.14 ± 0.04	0.14 ± 0.06	0.20 ± 0.00
74	34.04	compound 14	–	–	–	2382.02	-	0.24 ± 0.04	0.47 ± 0.22	0.25 ± 0.08	0.97 ± 0.86	0.42 ± 0.11	0.38 ± 0.22	0.20 ± 0.05	0.17 ± 0.12
					All		99.97 ± 0.05	99.97 ± 0.02	99.95 ± 0.02	99.96 ± 0.04	99.97 ± 0.03	99.92 ± 0.08	99.99 ± 0.02	99.99 ± 0.02	99.97 ± 0.02
				**Monoterpenes**	hydrocarbons		88.04 ± 0.89	88.00 ± 0.76	84.83 ± 2.71	88.79 ± 1.02	73.48 ± 2.98	84.08 ± 0.57	78.24 ± 2.28	84.49 ± 3.33	87.43 ± 2.02
					oxigenated		4.09 ± 0.08	5.92 ± 0.51	5.99 ± 0.46	5.87 ± 0.59	7.50 ± 0.81	5.42 ± 0.19	6.37 ± 0.38	5.56 ± 0.92	4.86 ± 0.51
				**Sesquiterpenes**	hydrocarbons		7.56 ± 0.80	3.08 ± 0.22	4.91 ± 0.79	2.00 ± 0.81	13.04 ± 0.67	5.63 ± 0.45	10.91 ± 0.42	6.41 ± 1.20	5.74 ± 0.49
					oxigenated		0.28 ± 0.01	0.21 ± 0.05	0.40 ± 0.12	0.26 ± 0.07	0.76 ± 0.12	0.89 ± 0.06	1.27 ± 0.16	0.75 ± 0.30	0.18 ± 0.16
				**Diterpenes**	hydrocarbons		0.00 ± 0.00	0.08 ± 0.04	0.11 ± 0.09	0.00 ± 0.00	0.36 ± 0.05	0.16 ± 0.03	0.28 ± 0.09	0.00 ± 0.00	0.05 ± 0.07
					oxigenated		0.00 ± 0.00	2.13 ± 0.27	1.79 ± 0.76	1.20 ± 0.18	2.92 ± 0.56	2.02 ± 0.39	2.14 ± 1.06	1.60 ± 0.56	1.26 ± 0.50
				**Not identified**			0.00 ± 0.00	0.54 ± 0.04	1.91 ± 0.71	1.84 ± 1.05	1.90 ± 0.81	1.73 ± 0.35	0.78 ± 0.26	1.18 ± 0.61	0.44 ± 0.35

a Odour descriptions were from Flavornet (www.flavornet.org). N.s. Not smelled.

b Compounds identification: RI and mass spectra mass spectra (MS) data compared against commercially available MS library NIST 23. tr, trace (<0.05%).

c RI: retention index experimentally determined on a ZB-5Plus column relative to the Rt of n-alkanes (C7-C40); compounds are listed in order of elution.

**Figure 2:**
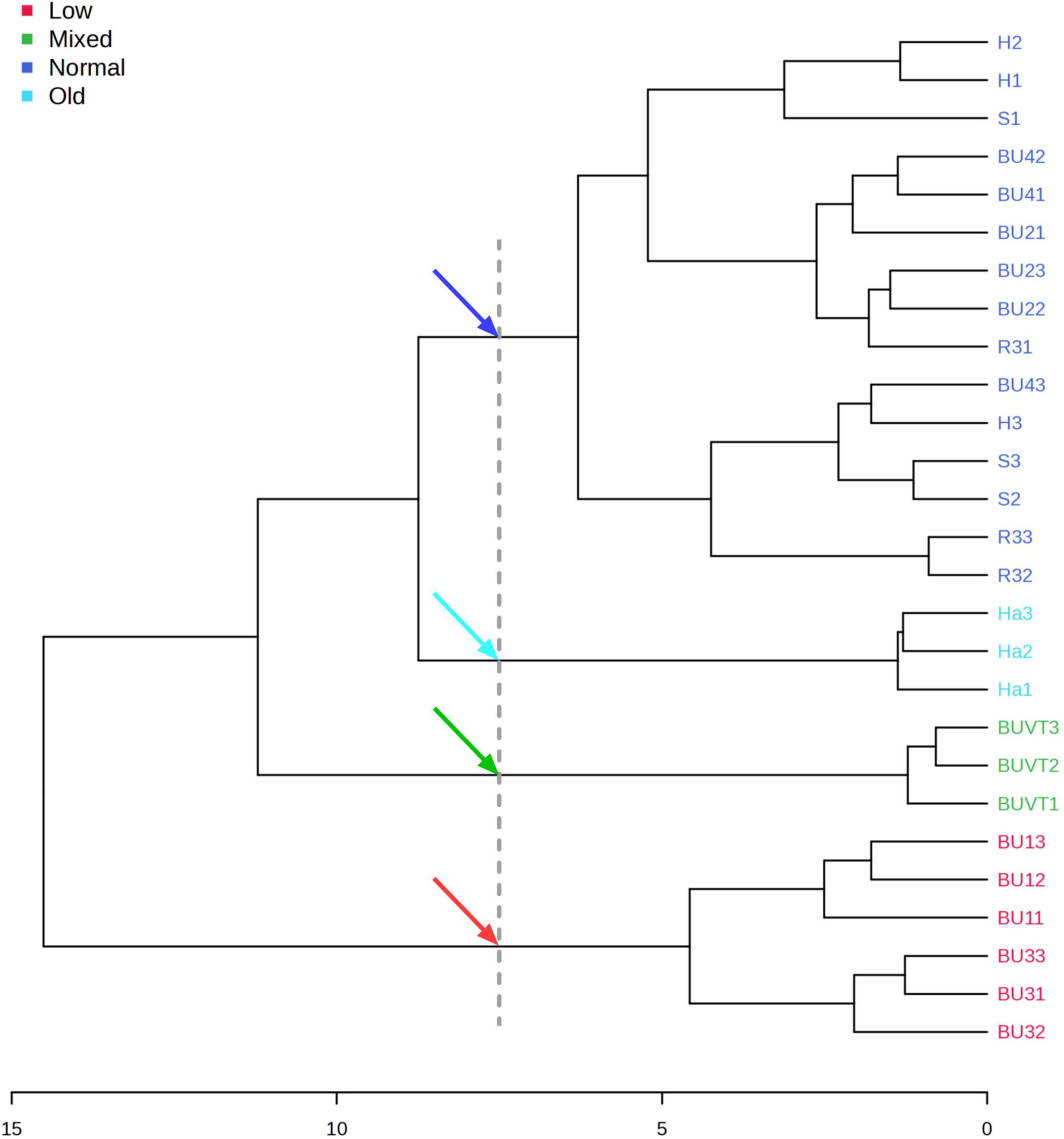
Dendrogram (hierarchical cluster analysis) representing the relationship of 27 oleoresins**’** essential oils obtained by Euclidian distance dissimilarity using the aggregation criterion of Ward**’**s method. Four main groups of samples (normal: normal composition according to specification; old: old sample; mixed: mixed species origin; low: low yield samples) were defined and colour-coded (represented by coloured arrows). The last digit of the sample code denotes the sample replicate number (1–3).

Columns are colour coded according to group assignments defined in **Figure 2**. Dark-blue represents normal samples, light-blue represents the old sample, red represents low yield samples, green represents the mixed species sample (group definition see section 3.3).

EOs with low yield (BU1, BU3) presented a lower amount of monoterpenes (80.98 ± 3.78%; 84.61 ± 2.66%) and the highest amount of sesquiterpenes (13.80 ± 0.80%; 12.18 ± 0.58%) as well as diterpenes (3.28 ± 0.61%; 2.42 ± 1.15%, respectively). The mixed oleoresin EO (BUVT) presented no diterpenes and the highest amount of β-pinene (**7**) (15.53 ± 0.17%), while the old oleoresin EO (Ha) had the highest amount of α-pinene (2) (72.39 ± 0.70%).

Visan et al. (2021) analysed young shoots from *L. decidua* and obtained another composition for the VOC. They found a lower amount of monoterpenes (hydrocarbons: 52.90 ± 0.53%; oxygenated: 4.01 ± 1.85%) in comparison to our findings (hydrocarbons: 73.48 ± 2.98% – 88.79 ± 1.02%; oxygenated: 4.09 ± 0.08% – 7.50 ± 0.81%). On the other hand, they obtained a higher amount of sesquiterpenes (hydrocarbons: 31.63 ± 6.53%; oxygenated: 6.69 ± 1.43%) when compared to the ones found for the oleoresins EO (hydrocarbons: 2.00 ± 0.81% – 13.04 ± 0.67%; oxygenated: 0.18 ± 0.16% – 1.27 ± 0.16%). The biosynthesis of terpenes in conifer oleoresins is initiated by the condensation of isopentenyl diphosphate (IPP) and dimethylallyl diphosphate (DMAPP), which originate from the methylerythritol phosphate (MEP) and mevalonic acid (MEV) pathways. The MEP pathway is responsible mainly for the biosynthesis of monoterpenes and diterpenes, while the MEV pathway is primarily used in sesquiterpenes biosynthesis. Transcripts for the MEP pathway are preferentially expressed in cortical resin ducts, the primary site of oleoresin biosynthesis (Celedon and Bohlmann, 2019). Our results demonstrated that the MEP pathway is prioritised in *L. decidua* oleoresins, since a higher content of monoterpenes and diterpenes was found in the oleoresins compared to the shoots.

Most compounds have been previously described for *L. decidua* (Batista et al., 2022), although a few new ones were identified in this study. These were α-pinene-oxide (17), α-phellandren-8-ol (23), δ–elemene (38), cyclosative (43), ylangene (44), and butylated hydroxytoluene (57). These compounds were described for the genus Pinus (Zhang and Wang, 2010; Nikolić et al., 2018; Thai et al., 2019; Ji and Ji, 2021; Mohamed et al., 2022), but none for the oleoresin. Those already described for *L. decidua* were mainly found in the bark, needle and wood samples only. The reported ones for the oleoresin were only 13-epimanool (70) (Norin, 1972; Mills, 1973; Norin and Winell, 1974; Bol'shakova et al., 2004; Salem et al., 2015; Thuerig et al., 2018; Dietemann et al., 2019), isopimarinal (71) (Bol'shakova et al., 2004; Bajer et al., 2020), and palustrinal (72) (Bol'shakova et al., 2004; Holmbom et al., 2008). In conclusion, all classes of volatile compounds were present in our samples. In contrast to previous reports, several new terpenes compounds were identified in the *L. decidua* oleoresin.

Other larch parts were investigated for their VOC (Kubeczka and Schultze, 1987; Weissmann and Reck, 1987; Holm and Hiltunen, 1997; Wajs et al., 2007; Garcia et al., 2017; Mofikoya et al., 2020; Visan et al., 2021). Weissmann and Reck (1987) obtained a similar composition of oleoresin VOC as we measured in our samples, with α-pinene (2) (76.4%), β-pinene (**7**) (7.7%), β-myrcene (8) (5.4%), 3-carene (10) (4.3%) as the major compounds. Although our results were similar to Weissmann and Reck (1987), the advantage of our study is a higher number of samples analysed, describing small variabilities within the same species. Kubeczka and Schultze (1987) examined three tree parts (needles, wood, bark) and observed a variability of their VOCs: α-pinene (2) was more concentrated in the wood (44.72%), followed by the bark (38.34%) and the needles (28.57%). On the contrary, 3-carene (10) was observed in higher concentration in the needles (19.19%), followed by the bark (4.80%) and the wood (2.78%). 3-carene (10) was observed to be in higher concentration in the needles, as described by Kubeczka and Schultze (1987) (19.19%), Holm and Hiltunen (1997) (5.8-21.6%), and (Weissmann and Reck, 1987) (24.2%), and therefore could be used as a chemical marker for the VOCs from *L. decidua*’s needles. Holm and Hiltunen (1997) described that the monoterpenes composition of leaf oils could be used as marker for genetic research and to edit issues related to population genetics of *Larix* species, which are characterised by high contents of α-pinene (2) and 3-carene (10). In conclusion, there are considerable differences with respect to VOC composition in different plant parts. Therefore, *L. decidua* oleoresins cannot be substituted by other plant parts to isolate their EOs.

### 3.3 Profiles of multivariate analyses

Unsupervised and supervised multivariate analyses were conducted to group and classify the differences between the analysed samples. After pre-processing and data normalisation, the final dataset used for the multivariate analysis consisted of 27 EOs samples x 74 features (relative abundance as % area).

Firstly, hierarchical cluster analysis (HCA) revealed four clearly defined groups (**Figure 2**). Group 1 was named low (low yield samples), group 2 mixed (mixed species oleoresin), group 3 old (the old sample kept in the institute), and group normal (oleoresins samples within specification). The corresponding colour-coding is defined and used in **Figure 2** and **Table 3**. Sample definitions are provided in sections 2.1 and 3.1. We then carried out a more detailed investigation with principal component analysis (PCA) to analyse the chemical pattern of these different groups.

Unsupervised PCA was applied to assess the composition of 9 oleoresin EOs and to identify a possible correlation between the various samples. The PCA score plot shows that principal component 1 (32.5%), principal component 2 (20.9%), and principal component 3 (12.4%) explained 65.8% of the data variance, which can reflect most of the information of the original data of the sample. The results of the PCA revealed four distinct groups (**Supplementary Figure 2**), corroborated by HCA analysis. The score plot demonstrates that the “mixed” group is composed only of one oleoresin EO, BUVT, the oleoresin named Venetian Turpentine composed of *Larix decidua*, *Abies alba*, *Pinus pinaster* and *Picea excelsa*’s oleoresins. The cluster “old” is formed only by one oleoresin EO (Ha), the oldest oleoresin studied, collected in the 1990s. The “low” group is characterised by two oleoresins’ EO, BU1 and BU3, which present the lowest EO yield and collection in the same year, 2020. The “normal” group comprises BU2, BU4, H, S, and R3, young oleoresins (collected 2018-2021) within the range of accepted EO yield. We concluded that PCA allows for a meaningful grouping of oleoresin samples based on their chemical fingerprint.

**Figure 3:**
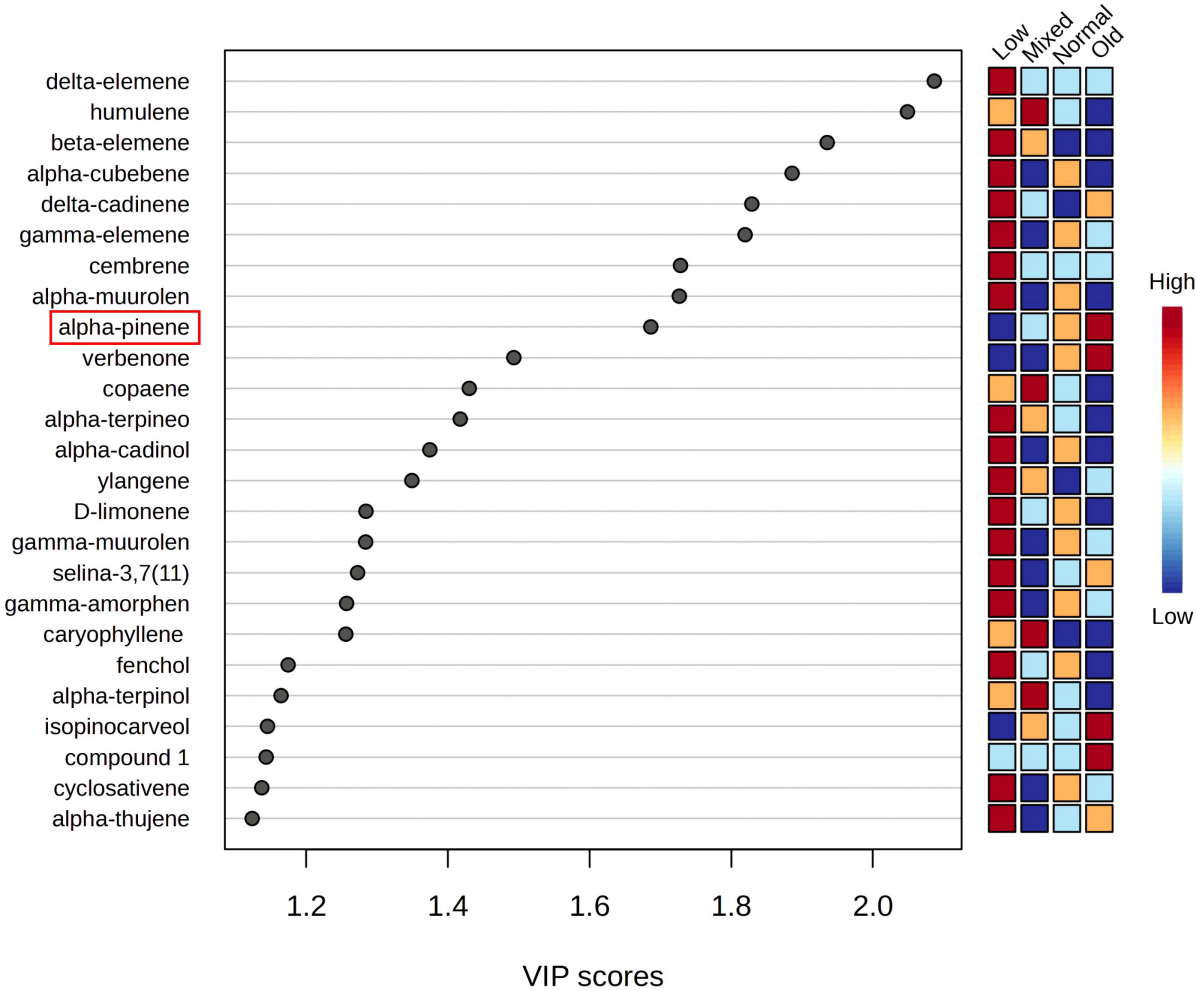
Variables important projection (VIPs) from PLS-DA of *L. decidua* oleoresins’ essential oils. The concentration was standardised at a range scale before analysis. Alpha-pinene is the most abundant volatile organic compound. Monitoring of the nine most important compounds (VIP scores > 1.6) is recommended to reliably group oleoresins. Groups of samples: normal: normal composition according to specification; old: old sample; mixed: mixed species origin; low: low yield samples (see **Figure 2**).

To understand the primary chemical compounds responsible for the initial separation observed in the PCA, a discriminant analysis (PLS-DA) was conducted to identify the main chemical constituents correlated to the clustering pattern observed in the scores plot through the 25 more critical variables in the projection (VIP). In **Figure 3**, for the old group (Ha), considering the VIP values, α-pinene (2) (VIP 1.69) and verbenone (29) (VIP 1.5) were the compounds with higher intensity in this group, followed by isopinocarveol (20). Concerning the normal group, the most important compounds for its differentiation are α-cubebene (41) (VIP 1.89) and γ-elemene (50) (VIP 1.82). The mixed group, composed of BUVT only, presented high intensity of the humulene (51) (VIP 2.05), copaene (45) (VIP 1.43), caryophyllene oxide (64) (VIP 1.26) and α-terpinolene (16) (VIP < 1.2). Lastly, the low group presented 17 high-intensity compounds among the 25 main VIPs in which the δ-elemene (38) (VIP 2.09) and β-elemene (46) (VIP 1.94) were the most important. We conclude that these most important compounds represent principal components, which can be used for grouping. This information is summarised in **Figure 3**. Consequently, monitoring of the nine most important compounds with VIP scores higher than 1.6 is sufficient to reliably group oleoresins and to detect, for example, adulterated or old samples.

**Figure 4:**
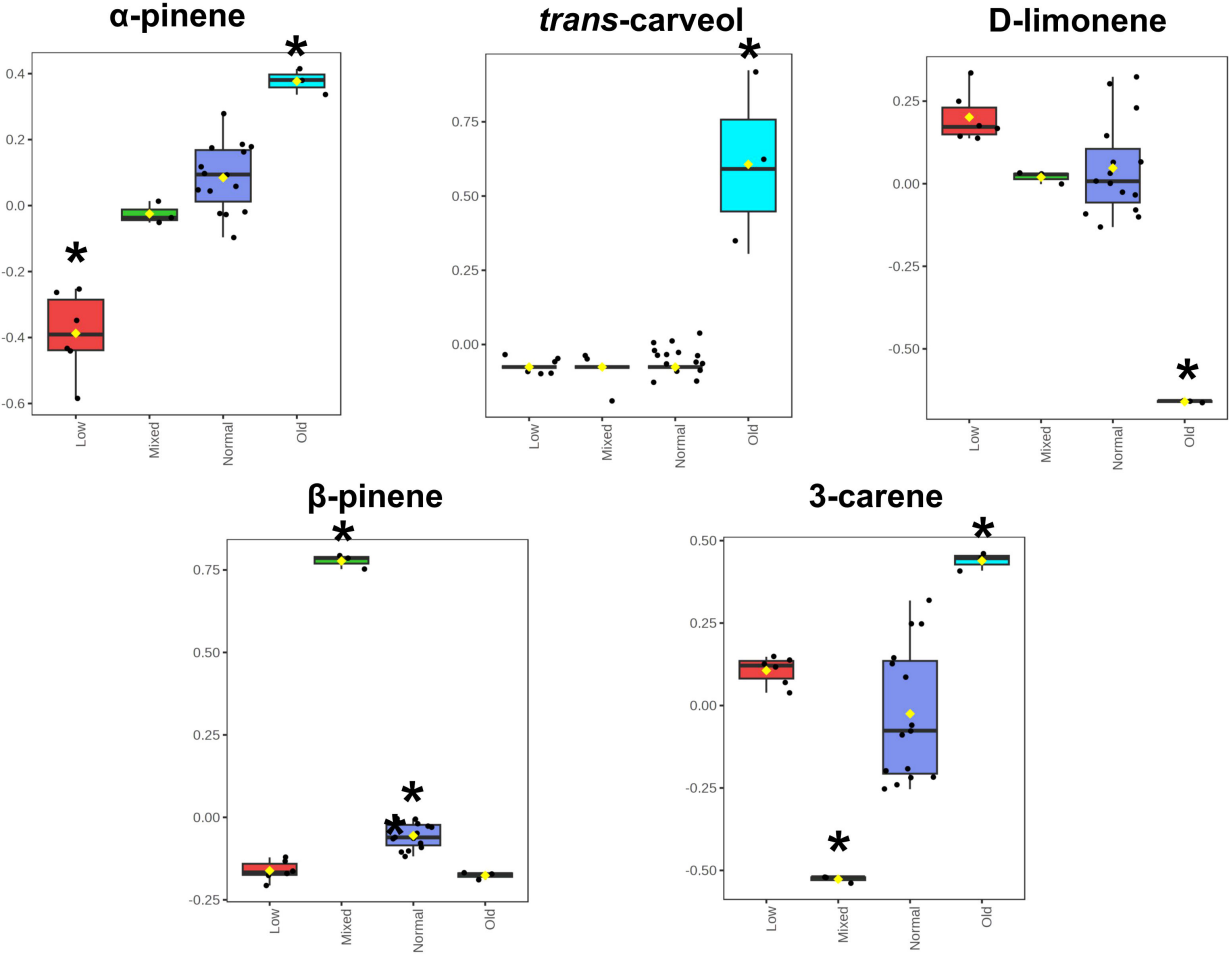
Selected chemical markers which can be used for grouping (normal: normal composition according to specification; old: old sample; mixed: mixed species origin; low: low yield samples, see **Figure 2**) based on biochemical considerations. Box plot representation of normalised concentrations of the respective compound and each group. Box-plot values represent median, 25% and 75% quartiles, and whiskers for minimum and maximum values. *p<0.05 one-way ANOVA with Tukey’s *post-hoc* analysis.

The question arises if, besides the statistical PCA approach, additional grouping markers can be defined based on biochemical considerations. In addition to α-pinene (2), verbenone (29) and isopinocarveol (20), which were more intense in the old group (**Supplementary Figure 3**), *trans*-carveol (30) could be used as a marker for the ageing or degradation of this species’ oleoresin EO (**Figure 4**). Supporting the idea of the degradation process, verbenone (29) and isopinocarveol (20) are degradation products of α-pinene (2) (Schrader et al., 2001), and D-limonene (14) was obtained in a low concentration in the old group, which is oxidised to *trans*-carveol (30) (Bouwmeester et al., 1998), found in higher amounts for this group (**Figure 4**), an indication of a degradation reaction (**Figure 4**). These oxidation reactions in the oleoresin may be related to daylight radiation and/or temperature influence in the storage process (Schrader et al., 2001).

For the mixed group (BUVT), 1,4-cineole (11) and α-longipinene (42) were present only in this group, the last previously described for *Abies alba* oleoresin (Zeneli et al., 2001). Although more samples should be considered to prove this idea, these compounds could be classed as adulterants of *L. decidua* oleoresin EOs since they were not found in pure samples nor described in the literature. β-pinene (7) was present in a higher concentration than the other groups (**Figure 4**), which the influence of different species can explain. Oleoresins from *Abies alba* presented similar proportions for α- and β-pinene (Zeneli et al., 2001) and equivalent amounts of β-pinene (**7**) (17.53-18.91%) were found for *Pinus pinaster* (Arrabal et al., 2002). Therefore, a higher concentration of β-pinene (7) for the mixed oleoresin is explained by the influence of the other species’ oleoresins in the sample (**Figure 4**). As previously discussed (section 3.2), 3-carene (10) is a vital chemical marker for the Larix oleoresin EOs. The PCA verified that a low concentration in the mixed group was obtained (**Figure 4**), resulting from an absence of this compound in *Abies alba* and *Pinus pinaster* oleoresins (Zeneli et al., 2001; Arrabal et al., 2002; Arrabal et al., 2005).

The compound in higher concentration in all samples was α-pinene (2) (VIP 1.69) but presented differences between the groups. Data (**Figure 4**) shows that its concentration decreased from the old, normal, mixed to the low group, statistically significant (p<0.05) except for the normal and mixed groups. α-pinene (2), a bicyclic monoterpene, is generated by the cyclization of geranyl pyrophosphate (GPP) by monoterpene synthases, specifically pinene synthases (I, II, III), responsible for the different stereochemistry (Loza-Tavera, 1999). It is found primarily in pine trees (coniferous) EOs and is the main secondary metabolite in many conifer-derived EOs, the one responsible for the characteristic smell of pine trees (Salehi et al., 2019; Allenspach and Steuer, 2021; Nyamwihura and Ogungbe, 2022). VOCs, such as α-pinene (2) and β-pinene (7), possess influence on plants defences, working as plant-to-plant signalling, leading to a systematic acquired resistance (Riedlmeier et al., 2017) and helping plants to communicate and to fight against parasites, such as fungi and bacteria (Nyamwihura and Ogungbe, 2022). Their medical properties are described for several purposes since they possess therapeutic potential as anticoagulant, antitumoral, gastroprotective, anxiolytic, neuroprotective, antimicrobial, antimalarial, insecticidal and larvicidal, antifungal, anti-inflammatory, analgesic products, among others (Salehi et al., 2019; Allenspach and Steuer, 2021; Nyamwihura and Ogungbe, 2022).

Other VOCs that also appear to bear some importance in the analysed oleoresins are D-limonene (14), α-terpineol (27), β-myrcene (8), and 3-carene (10). They are not only important for the typical conifer fragrance, but are involved in intraspecific host-finding pheromones communication, play a major defensive role against insects and pathogens, and are important for cultures due to economic reasons and pharmacological properties (Langenheim, 2003). An interesting review compared the activity of D-limonene (14) and perillyl alcohol, a hydroxylated analogue of D-limonene (14), on breast cancer in human trials. They concluded among 5 studies that D-limonene (14) possessed better tolerability and chemopreventive properties than the perillyl alcohol, but further well-designed studies should be carried out (Chebet et al., 2021). A recent study described the potential of D-limonene (14) as anti-SARS-CoV-2 candidate, since it possesses similarities in structure with the thymidine of SARS-CoV-2 genome and low cytotoxic effects in MRC-5 (fibroblast) and HaCaT (keratinocyte) cell lines (Correa et al., 2023). Although several *in vivo* studies have described the potential of D-limonene (14), limited data exists for its tolerability and safety in humans (Anandakumar et al., 2021). Khaleel et al. (2018) described in a review several biological properties of α-terpineol (27), such as antihypertensive, antiproliferative, antiulcer and insecticidal. The most important activity correlated to α-terpineol (27) is its anti-nociceptive activity, with highly analgesic effects in mice, mainly due to inhibition of pro-inflammatory molecules release. Oil from *Eucalyptus globulus* as well as α-terpineol (27) demonstrated anti-parasitic effects against *Pediculus humanus capitis*, an ectoparasite confined in human scalp and hair (Yang et al., 2004). In an *in vitro* study, α-terpineol (27) inhibited the growth and induced cell death in tumour cells via inhibition of NF-κB activity, among other mechanisms (Hassan et al., 2010). A recent publication described that the biological properties of β-myrcene (8) are coupled with its non-allergic, non-toxic and antimutagenic activities. It has anxiolytic and sedative effect; it acts as an antioxidant agent, which is accountable for prevention of ageing and degenerative diseases; its powerful anti-inflammatory activity *in vitro* lies mainly through PGE-2; the analgesic effects are central and peripheral (Surendran et al., 2021). In addition, McDougall and McKenna (2022) demonstrated *in vivo* the reduction of joint pain and inflammation in rats, suggesting the potential of β-myrcene (8) to reduce chronic arthritis pain and inflammation. Lastly, 3-carene (10) was proven to be the most prominent agent against dermatophytes and could be used as an antifungal compound (Cavaleiro et al., 2006). In addition, another study showed that 3-carene (10), among other compounds, possessed the broadest spectrum of activity against fungi and gram-positive bacteria, which could be used as antimicrobial agent and to prevent aflatoxin contamination in foods (Cosentino et al., 2003).

Keeping the oleoresins in closed packages at room temperature for up to three years prevented degradation. Thus, storage under these conditions does not seem to influence the quality of the samples. Further studies should be performed to verify when degradation starts as a function of storage methods. It remains to be elucidated to which degree these metabolites change in relation to the time of collection, geographical location, and seasonal variation. We conclude that further analysis is necessary to decide whether they are, with exception of α-pinene (2), reliable markers for grouping, such as evaluated, into “normal” (normal composition according to specification), “old” (old sample), “mixed” (mixed species origin), and “low” (low yield samples).

## 4 Conclusion

Chemical fingerprinting based on GC-MS analysis is a prerequisite to group oleoresins and to detect preparations, which are not suitable for pharmaceutical applications. In the present work, strategies are provided to carry out this task. Chemical variances in essential oils observed in nine samples of oleoresins obtained from four companies and collection sites show the importance of standardisation and storage to guarantee reproducible chemical composition in production batches. Information on geographic location and collection date is mandatory. Care should be taken to avoid preparations adulterated by addition of volatile organic compounds from preparations other than oleoresins or other plant species. In addition, we identified for the first time α-pinene-oxide, α-phellandren-8-ol, δ–elemene, cyclosative, ylangene, and butylated hydroxytoluene in *Larix* oleoresin and suggested possible adulterants (1,4-cineole and α-longipinene) and compounds related to ageing (*trans*-carveol). The question arises if alternative analytical technologies could be used to increase the number of detected metabolites. With this respect, headspace solid-phase microextraction would avoid potential loss of compound during hydrodistillation and accelerate the analytical procedure.

## Data availability statement

The raw data supporting the conclusions of this article will be made available by the authors, without undue reservation.

## Author contributions

JB: Data curation, Formal analysis, Investigation, Methodology, Writing – original draft, Conceptualization, Writing – review & editing. MM: Data curation, Formal analysis, Writing – review & editing. CH: Conceptualization, Supervision, Writing – review & editing. JM: Resources, Supervision, Writing – review & editing. JH: Conceptualization, Supervision, Writing – review & editing. SB: Conceptualization, Funding acquisition, Resources, Supervision, Visualization, Writing – review & editing. FB: Conceptualization, Supervision, Writing – review & editing.

## References

[B1] Allenspach M. Steuer C. (2021). alpha-Pinene: A never-ending story. Phytochemistry 190, 112857. doi: 10.1016/j.phytochem.2021.112857 34365295

[B2] Anandakumar P. Kamaraj S. Vanitha M. K. (2021). D-limonene: A multifunctional compound with potent therapeutic effects. J. Food Biochem. 45 (1), e13566. doi: 10.1111/jfbc.13566 33289132

[B3] Arrabal C. Cortijo M. de Simón B. F. García-Vallejo M. C. Cadahía E. (2002). *Pinus pinaster* oleoresin in plus trees. Holzforschung 56 (3), 261–266. doi: 10.1515/hf.2002.043

[B4] Arrabal C. Cortijo M. de Simón B. F. García Vallejo M. C. Cadahía E. (2005). Differentiation among five Spanish Pinus pinaster provenances based on its oleoresin terpenic composition. Biochem. Systematics Ecol. 33 (10), 1007–1016. doi: 10.1016/j.bse.2005.03.003

[B5] Atanasov A. G. Zotchev S. B. Dirsch V. M. International Natural Product Sciences, T Supuran C. T. (2021). Natural products in drug discovery: advances and opportunities. Nat. Rev. Drug Discovery 20 (3), 200–216. doi: 10.1038/s41573-020-00114-z 33510482 PMC7841765

[B6] Ayub M. A. Choobkar N. Hanif M. A. Abbas M. Ain Q. U. Riaz M.. (2022). Chemical composition and biological potential of *Pinus roxburghii oleoresin* essential oils extracted by steam distillation, superheated steam, and supercritical. Res. Square. doi: 10.21203/rs.3.rs-2202313/v1 37199441

[B7] Bajer T. Šulc J. Ventura K. Bajerová P. (2020). Volatile compounds fingerprinting of larch tree samples for Siberian and European larch distinction. Eur. J. Wood Wood Products 78 (2), 393–402. doi: 10.1007/s00107-020-01498-w

[B8] Batista J. V. C. Uecker A. Holandino C. Boylan F. Maier J. Huwyler J.. (2022). A scoping review on the therapeutic potential of resin from the species *larix decidua* mill. [Pinaceae] to treat ulcerating wounds. Front. Pharmacol. 13. doi: 10.3389/fphar.2022.895838 PMC9204203 35721139

[B9] Bol'shakova V. I. Demenkova L. I. Shmidt É.N. Pentegova V. A. (2004). Neutral diterpenoids of oleoresins of five species of conifers of *Transcarpathia* . Chem. Natural Compounds 24 (6), 691–694. doi: 10.1007/bf00598185

[B10] Bouwmeester H. J. Gershenzon J. Konings M. C. Croteau R. (1998). Biosynthesis of the monoterpenes limonene and carvone in the fruit of caraway. I. Demonstration Of enzyme activities and their changes with development. Plant Physiol. 117 (3), 901–912. doi: 10.1104/pp.117.3.901 9662532 PMC34944

[B11] Cavaleiro C. Pinto E. Goncalves M. J. Salgueiro L. (2006). Antifungal activity of Juniperus essential oils against *dermatophyte*, *Aspergillus* and *Candida* strains. J. Appl. Microbiol. 100 (6), 1333–1338. doi: 10.1111/j.1365-2672.2006.02862.x 16696681

[B12] Celedon J. M. Bohlmann J. (2019). Oleoresin defenses in conifers: chemical diversity, terpene synthases and limitations of oleoresin defense under climate change. New Phytol. 224 (4), 1444–1463. doi: 10.1111/nph.15984 31179548

[B13] Chebet J. J. Ehiri J. E. McClelland D. J. Taren D. Hakim I. A. (2021). Effect of d-limonene and its derivatives on breast cancer in human trials: a scoping review and narrative synthesis. BMC Cancer 21 (1), 902. doi: 10.1186/s12885-021-08639-1 34362338 PMC8349000

[B14] Correa A. N. R. Weimer P. Rossi R. C. Hoffmann J. F. Koester L. S. Suyenaga E. S.. (2023). Lime and orange essential oils and d-limonene as a potential COVID-19 inhibitor: Computational, in chemico, and cytotoxicity analysis. Food Biosci. 51, 102348. doi: 10.1016/j.fbio.2022.102348 36597499 PMC9801698

[B15] Cosentino S. Barra A. Pisano B. Cabizza M. Pirisi F. M. Palmas F. (2003). Composition and antimicrobial properties of Sardinian Juniperus essential oils against foodborne pathogens and spoilage microorganisms. J. Food Prot 66 (7), 1288–1291. doi: 10.4315/0362-028x-66.7.1288 12870766

[B16] Da Ronch F. C. Tinner W. de Rigo D. (2016). “ *Larix decidua* and other larches in Europe: distribution, habitat, usage and threats,” in European atlas of forest tree species. Eds. San-Miguel-Ayanz D. Caudullo G. Houston Durrant T. Mauri A. (Luxembourg: Publ. Off. EU), e01e492+.

[B17] Dietemann P. Miller K.v. Höpker C. Baumer U. (2019). On the use and differentiation of resins from pinaceae species in european artworks based on written sources, reconstructions and analysis. Stud. Conserv. 64 (sup1), S62–S73. doi: 10.1080/00393630.2019.1568678

[B18] Dzobo K. (2022). “The role of natural products as sources of therapeutic agents for innovative drug discovery,” in Comprehensive pharmacology. Ed. Kenakin T. (Amsterdam: Elsevier), 408–422.

[B19] Figueiredo A. C. Barroso J. G. Pedro L. G. Scheffer J. J. C. (2008). Factors affecting secondary metabolite production in plants: volatile components and essential oils. Flavour Fragrance J. 23 (4), 213–226. doi: 10.1002/ffj.1875

[B20] Franz C. Novak J. (2015). “Sources of essential oils,” in Handbook of essential oils. Eds. Can Başer K. H. Buchbauer G.. (London: CRC Press), 43–86.

[B21] Garcia G. Garcia A. Gibernau M. Bighelli A. Tomi F. (2017). Chemical compositions of essential oils of five introduced conifers in Corsica. Nat. Prod Res. 31 (14), 1697–1703. doi: 10.1080/14786419.2017.1285299 28278672

[B22] HAB. (2014). Terebinthina laricina. Stuttgart: Deutscher Apotheker Verlag.

[B23] Harrewijn P. van Oosten A. M. Piron P. G. M. (2000). Natural terpenoids as messengers. Dordrecht: Springer.

[B24] Hassan S. B. Gali-Muhtasib H. Göransson H. Larsson R. (2010). Alpha Terpineol: A Potential Anticancer Agent which Acts through Suppressing NF-κB Signalling. Anticancer Res. 30 (6), 1911–1919. 20651334

[B25] Holm Y. Hiltunen R. (1997). Variation and inheritance of monoterpenes inLarix species. Flavour Fragrance J. 12 (5), 335–339. doi: 10.1002/(sici)1099-1026(199709/10)12:5<335::Aid-ffj664>3.0.Co;2-i

[B26] Holmbom T. Reunanen M. Fardim P. (2008). Composition of callus resin of Norway spruce, Scots pine, European larch and Douglas fir. hfsg 62 (4), 417–422. doi: 10.1515/hf.2008.070

[B27] Ji W. Ji X. (2021). Comparative analysis of volatile terpenes and terpenoids in the leaves of pinus species-A potentially abundant renewable resource. Molecules 26 (17). doi: 10.3390/molecules26175244 PMC8433728 34500678

[B28] Karimian V. Sepehry A. Barani H. Ebrahimi S. N. Mirjalili M. H. (2020). Productivity, essential oil variability and antioxidant activity of Ferula assa-foetida L. oleo-gum-resin during the plant exploitation period. J. Essential Oil Res. 32 (6), 545–555. doi: 10.1080/10412905.2020.1794988

[B29] Khaleel C. Tabanca N. Buchbauer G. (2018). α-Terpineol, a natural monoterpene: A review of its biological properties. Open Chem. 16 (1), 349–361. doi: 10.1515/chem-2018-0040

[B30] Kubeczka K. H. Schultze W. (1987). Biology and chemistry of conifer oils. Flavour Fragrance J. 2 (4), 137–148. doi: 10.1002/ffj.2730020402

[B31] Lagoni N. (2012) Vom Lärchenharz zum Terpentin bis Lärchenöl. Wissen. Available at: https://www.waldwissen.net/de/waldwirtschaft/nebennutzung/waldprodukte/laerchenharz (Accessed 02.10.2023).

[B32] Langenheim J. H. (2003). Plant resins: chemistry, evolution, ecology, and ethnobotany (Portland: Timber Press).

[B33] Loza-Tavera H. (1999). Monoterpenes in essential oils. Biosynthesis and properties. Adv. Exp. Med. Biol. 464, 49–62. doi: 10.1007/978-1-4615-4729-7_5 10335385

[B34] McDougall J. J. McKenna M. K. (2022). Anti-inflammatory and analgesic properties of the cannabis terpene myrcene in rat adjuvant monoarthritis. Int. J. Mol. Sci. 23 (14). doi: 10.3390/ijms23147891 PMC9319952 35887239

[B35] Melo M. N. O. Ochioni A. C. Zancan P. Oliveira A. P. Grazi M. Garrett R.. (2022). Viscum album mother tinctures: Harvest conditions and host trees influence the plant metabolome and the glycolytic pathway of breast cancer cells. Front. Pharmacol. 13. doi: 10.3389/fphar.2022.1027931 PMC9662615 36386174

[B36] Mills J. S. (1973). Diterpenes of larix oleoresins. Phytochemistry 12 (10), 2407–2412. doi: 10.1016/0031-9422(73)80447-9

[B37] Mofikoya O. O. Makinen M. Janis J. (2020). Chemical fingerprinting of conifer needle essential oils and solvent extracts by ultrahigh-resolution fourier transform ion cyclotron resonance mass spectrometry. ACS Omega 5 (18), 10543–10552. doi: 10.1021/acsomega.0c00901 32426612 PMC7227056

[B38] Mohamed M. E. Tawfeek N. Elbaramawi S. S. Fikry E. (2022). Agathis robusta Bark Essential Oil Effectiveness against COVID-19: Chemical Composition, In Silico and *In Vitro* Approaches. Plants (Basel) 11 (5). doi: 10.3390/plants11050663 PMC8912836 35270131

[B39] Mohtashami R. Hashem-Dabaghian F. Nabati F. Qaderi A. Kianbakht S. (2023). Efficacy of topical oleoresin of *Pistacia atlantica* L. subspecies kurdica for symptomatic relief of knee osteoarthritis: A randomized double-blinded controlled trial. J. Herbal Med. 38. doi: 10.1016/j.hermed.2023.100638

[B40] National Institute of Standards and Technology (NIST). (2023) NIST standard reference database number 69, 2023. doi: 10.18434/T4D303

[B41] Nikolić B. Todosijević M. Ratknić M. Đorđević I. Stanković J. Cvetković M.. (2018). Terpenes and n-Alkanes in Needles of *Pinus cembra* . Natural Product Commun. 13 (8), 1035–1037. doi: 10.1177/1934578x1801300828

[B42] Norin T. (1972). Some aspects of the chemistry of the order Pinales. Phytochemistry 11 (4), 1231–1242. doi: 10.1016/s0031-9422(00)90069-4

[B43] Norin T. Winell B. (1974). Neutral constituents of *Larix decidua* bark. Phytochemistry 13 (7), 1290–1292. doi: 10.1016/0031-9422(74)80121-4

[B44] Nyamwihura R. J. Ogungbe I. V. (2022). The pinene scaffold: its occurrence, chemistry, synthetic utility, and pharmacological importance. RSC Adv. 12 (18), 11346–11375. doi: 10.1039/d2ra00423b 35425061 PMC9003397

[B45] Rasgado-Bonilla F. A. Soto-Hernández R. M. Conde-Martínez V. Vibrans H. Cibrián-Tovar D. (2016). Variación estacional en la composición química de resinas y aceites esenciales de Liquidambar styraciflua de Hidalgo, México. Botanical Sci. 94 (2), 331–344. doi: 10.17129/botsci.286

[B46] Riedlmeier M. Ghirardo A. Wenig M. Knappe C. Koch K. Georgii E.. (2017). Monoterpenes support systemic acquired resistance within and between plants. Plant Cell 29 (6), 1440–1459. doi: 10.1105/tpc.16.00898 28536145 PMC5502447

[B47] Salehi B. Upadhyay S. Erdogan Orhan I. Kumar Jugran A. Jayaweera S. L. D. Dias D. A.. (2019). Therapeutic Potential of alpha- and beta-Pinene: A Miracle Gift of Nature. Biomolecules 9 (11). doi: 10.3390/biom9110738 PMC6920849 31739596

[B48] Salem M. Z. M. Zeidler A. Böhm M. Mohamed M. E. A. Ali H. M. (2015). GC/MS Analysis of Oil Extractives from Wood and Bark of Pinus sylvestris, Abies alba, Picea abies, and *Larix decidua* . BioResources 10 (4), 7725–7737. doi: 10.15376/biores.10.4.7725-7737

[B49] Sarria-Villa R. A. Gallo-Corredor J. A. Benitez-Benitez R. (2021). Characterization and determination of the quality of rosins and turpentines extracted from Pinus oocarpa and Pinus patula resin. Heliyon 7 (8), e07834. doi: 10.1016/j.heliyon.2021.e07834 34485729 PMC8405890

[B50] Schrader W. Geiger J. Klockow D. Korte E. H. (2001). Degradation of alpha-pinene on Tenax during sample storage: effects of daylight radiation and temperature. Environ. Sci. Technol. 35 (13), 2717–2720. doi: 10.1021/es0002722 11452597

[B51] Sharmeen J. B. Mahomoodally F. M. Zengin G. Maggi F. (2021). Essential oils as natural sources of fragrance compounds for cosmetics and cosmeceuticals. Molecules 26 (3). doi: 10.3390/molecules26030666 PMC7865210 33514008

[B52] Sukarno A. Hardiyanto E. Marsoem S. Na'iem M. (2015). Oleoresin production, turpentine yield and components of Pinus merkusii from various Indonesian Provenances. J. Trop. For. Sci. 27, 136–141.

[B53] Surendran S. Qassadi F. Surendran G. Lilley D. Heinrich M. (2021). Myrcene-what are the potential health benefits of this flavouring and aroma agent? Front. Nutr. 8. doi: 10.3389/fnut.2021.699666 PMC8326332 34350208

[B54] Thai T. H. Hien N. T. Diep L. N. Paoli M. Casanova J. Tomi F. (2019). Chemical composition of needle, cone, and branch oils from Vietnamese pinus cernua. Natural Product Commun. 14 (5), 53–60. doi: 10.1177/1934578x19850992

[B55] The_World_Flora (2023) Larix decidua (L.) Mill (The World Flora). Available at: https://wfoplantlist.org/plant-list/taxon/wfo-0000443338-2023-06?page=1 (Accessed 16 November 2023).

[B56] Thuerig B. James E. E. Scharer H. J. Langat M. K. Mulholland D. A. Treutwein J.. (2018). Reducing copper use in the environment: the use of larixol and larixyl acetate to treat downy mildew caused by Plasmopara viticola in viticulture. Pest Manag Sci. 74 (2), 477–488. doi: 10.1002/ps.4733 28905481

[B57] Tongnuanchan P. Benjakul S. (2014). Essential oils: extraction, bioactivities, and their uses for food preservation. J. Food Sci. 79 (7), R1231–R1249. doi: 10.1111/1750-3841.12492 24888440

[B58] Tropicos (2023) Larix decidua Mill (Tropicos). Available at: https://www.tropicos.org/name/24900158 (Accessed 16 November 2023).

[B59] Visan D. C. Oprea E. Radulescu V. Voiculescu I. Biris I. A. Cotar A. I.. (2021). Original contributions to the chemical composition, microbicidal, virulence-arresting and antibiotic-enhancing activity of essential oils from four coniferous species. Pharm. (Basel) 14 (11). doi: 10.3390/ph14111159 PMC8617773 34832941

[B60] Wajs A. Pranovich A. Reunanen M. Willför S. Holmbom B. (2007). Headspace-SPME analysis of the sapwood and heartwood of *Picea abies*, *pinus sylvestris* and *Larix decidua* . J. Essential Oil Res. 19 (2), 125–133. doi: 10.1080/10412905.2007.9699244

[B61] Weissmann v.G. Reck S. (1987). Identifizierung von Hybridlärchen mit Hilfe chemischer Merkmale. Silvae Genetica 36, 5.

[B62] Yang Y. C. Choi H. Y. Choi W. S. Clark J. M. Ahn Y. J. (2004). Ovicidal and adulticidal activity of Eucalyptus globulus leaf oil terpenoids against Pediculus humanus capitis (Anoplura: Pediculidae). J. Agric. Food Chem. 52 (9), 2507–2511. doi: 10.1021/jf0354803 15113148

[B63] Yu N. Li Q. Yang J. Yin G. Li R. Zou W. (2020). Variation in oleoresin yield and anatomical traits among Sindora glabra populations in Hainan, China. Trees 34 (5), 1323–1334. doi: 10.1007/s00468-020-02000-y

[B64] Zeneli G. Tsitsimpikou C. Petrakis P. V. Naxakis G. Habili D. Roussis V. (2001). Foliar and cortex oleoresin variability of silver fir (Abies alba Mill.) in Albania. Z Naturforsch. C J. Biosci. 56 (7-8), 531–539. doi: 10.1515/znc-2001-7-810 11531086

[B65] Zhang Y. Wang Z. (2010). Comparative analysis of essential oil components of two pinus species from taibai mountain in China. Natural Product Commun. 5 (8), 1295–1298. doi: 10.1177/1934578x1000500831 20839639

