# Supplementary material for: Characterization of Larix decidua Mill. (Pinaceae) oleoresin’s essential oils composition using GC-MS
Source: Front Plant Sci. 2024 Jan 8;14:1331894. doi: 10.3389/fpls.2023.1331894 (PMC10801252; doi:10.3389/fpls.2023.1331894)
Supplement: Supplementary file 1 [file DataSheet_1.docx]

***Supplementary Material***

**Characterization of *Larix decidua* Mill. (Pinaceae) oleoresin’s essential oils composition using GC-MS**

**João V.C. Batista^1,2^, Michelle Nonato de Oliveira Melo^3^, Carla Holandino^1,3*^, Jakob Maier^1^, Jörg Huwyler^2*^, Stephan Baumgartner^1,4,5*^, Fabio Boylan^6*^**

^1^ Hiscia Institute, Society for Cancer Research, Arlesheim, Switzerland

^2^ Department of Pharmaceutical Sciences, Division of Pharmaceutical Technology, University of Basel, Basel, Switzerland

^3^ Departamento de Fármacos e Medicamentos, Faculdade de Farmácia, Universidade Federal do Rio de Janeiro, Rio de Janeiro, Brasil

^4^ Institute of Integrative Medicine, University of Witter/Herdecke, Witten, Germany

^5^ Institute of Complementary and Integrative Medicine, University of Bern, Bern, Switzerland

^6^ School of Pharmacy and Pharmaceutical Sciences, Trinity Biomedical Sciences Institute, Trinity Natural Products Research Centre, Trinity College Dublin, Dublin, Ireland

*** Correspondence:**Prof. Dr. Carla Holandino, Prof. Dr. Jörg Huwyler, Prof. Dr. Stephan Baumgartner, Prof. Dr. Fabio Boylan

E-mail: cholandino@gmail.com; joerg.huwyler@unibas.ch; stephan.baumgartner@unibe.ch; fabio.boylan@tcd.ie

**Figures**


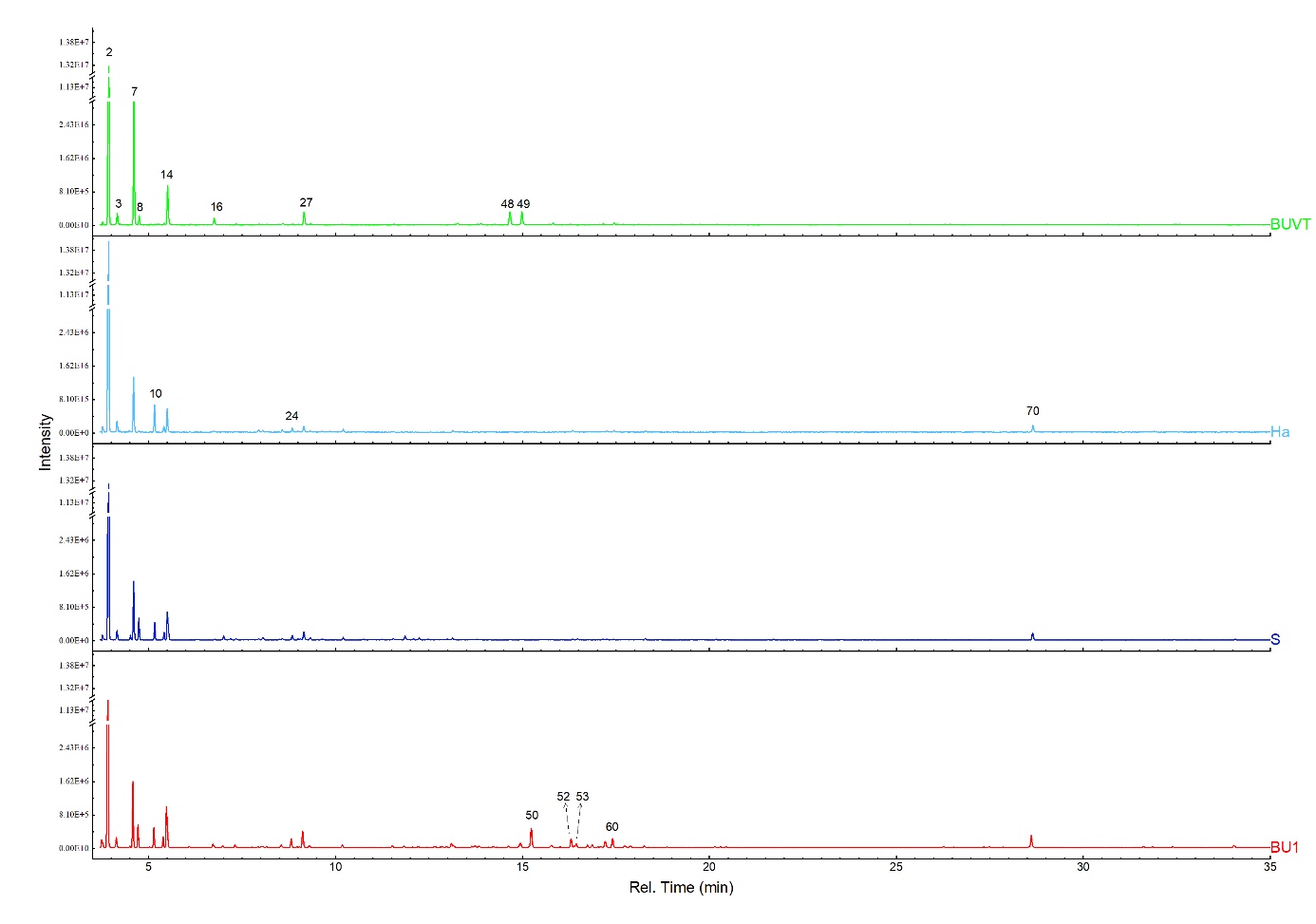


**Figure S1**. Typical GC-MS chromatograms of essential oils of *Larix decidua* Mill. oleoresins from different manufacturers. Compounds were eluted using a ZB-5Plus column. Enumerated compounds are present at a concentration higher than 1% in at least one oleoresin EO. All the compounds are described in **Table 3**.


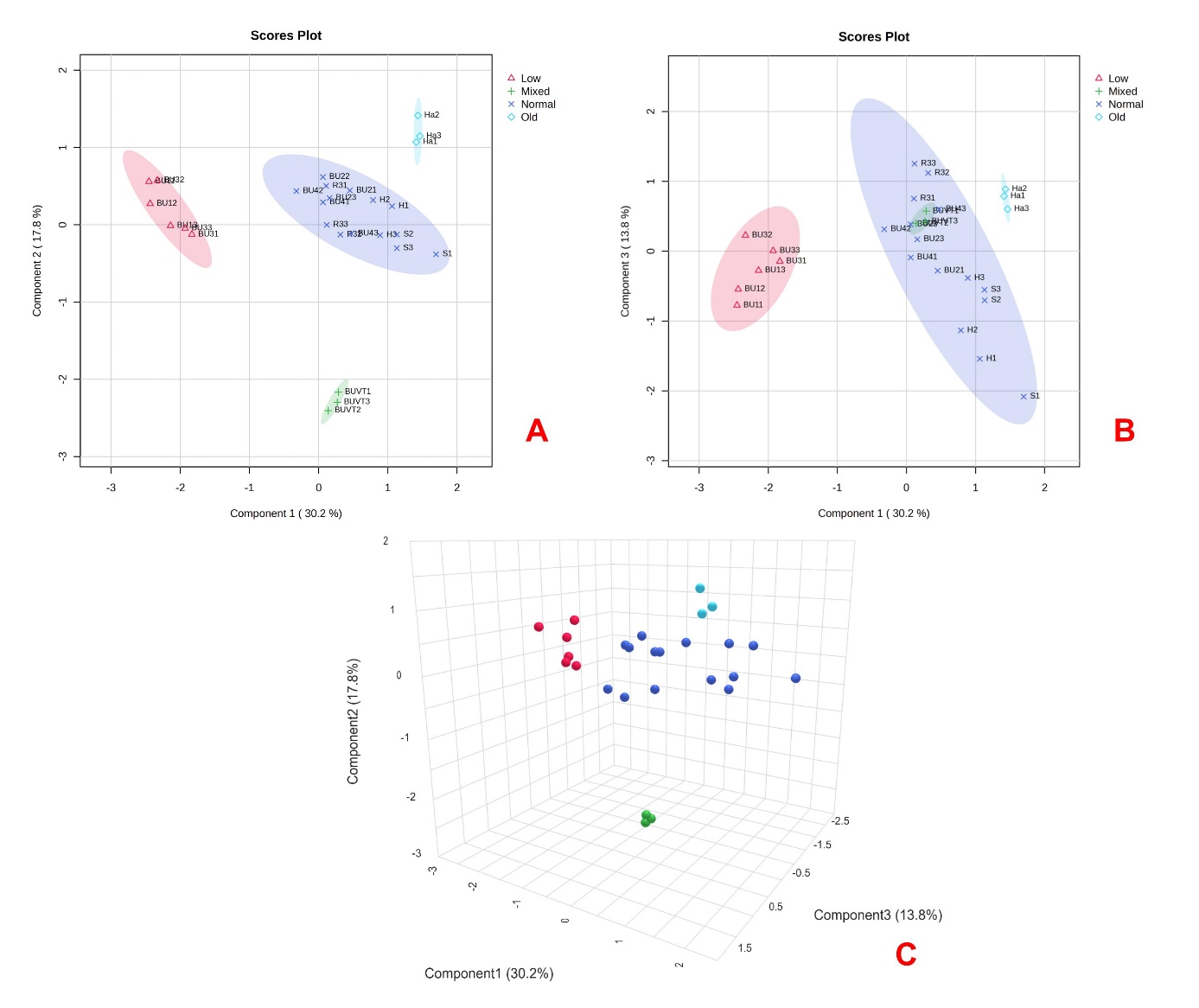


**Figure S2**. Multivariate analysis from GC-MS data of *Larix decidua* oleoresins' essential oils. Principal component analysis applied to volatile organic compounds obtained by distillation (n=3) of each oleoresin. A- PC1 x PC2 score plot; B- PC1 x PC3 score plot; C- 3D PC1 x PC2 x PC3 score plot. Samples are colour-coded according to group definitions (see Figure 2).


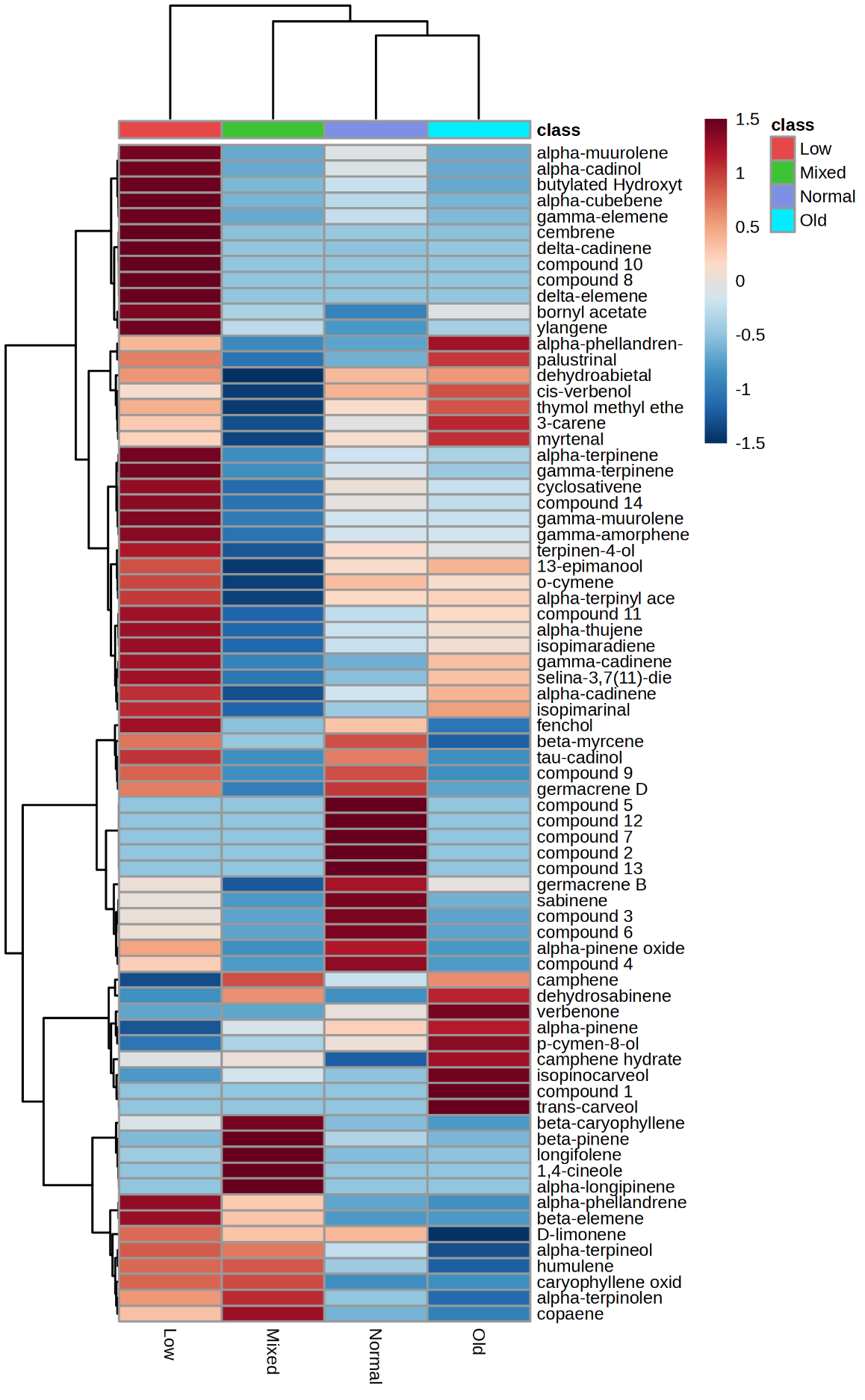


**Figure S3**. Heatmap for 74 volatile organic compounds of the four oleoresins groups. Samples are colour-coded according to group definitions (see **Figure 3**).
